# OsmiR167a‐targeted auxin response factors modulate tiller angle via fine‐tuning auxin distribution in rice

**DOI:** 10.1111/pbi.13360

**Published:** 2020-03-04

**Authors:** Yan Li, Jiali Li, Zhihui Chen, Yi Wei, Yanhua Qi, Changyin Wu

**Affiliations:** ^1^ National Key Laboratory of Crop Genetic Improvement National Center of Plant Gene Research (Wuhan) Huazhong Agricultural University Wuhan China; ^2^ College of Life Sciences Hubei University Wuhan China; ^3^ Institute of Rice Research Guizhou Academy of Agricultural Sciences Guiyang China; ^4^ State Key Laboratory of Plant Physiology and Biochemistry College of Life Sciences Zhejiang University Hangzhou China

**Keywords:** OsmiR167a, auxin response factors, auxin asymmetric distribution, tiller angle, rice

## Abstract

Rice tiller angle determines plant growth density and further contributes grain production. Although a few genes have been characterized to regulate tiller angle in rice, the molecular mechanism underlying the control of tiller angle via microRNA is poorly understood. Here, we report that rice tiller angle is controlled by OsmiR167a‐targeted auxin response factors *OsARF12*, *OsARF17* and Os*ARF25*. In the overexpression of *OsMIR167a* plants, the expression of *OsARF12*, *OsARF17* and Os*ARF25* was severely repressed and displayed larger tiller angle as well as the *osarf12/osarf17* and *osarf12/ osarf25* plants. In addition, those plants showed compromised abnormal auxin distribution and less sensitive to gravity. We also demonstrate that *OsARF12*, *OsARF17* and Os*ARF25* function redundantly and might be involved in *HSFA2D* and *LAZY1‐*dependent asymmetric auxin distribution pathway to control rice tiller angle. Our results reveal that OsmiR167a represses its targets, *OsARF12*, *OsARF17* and Os*ARF25*, to control rice tiller angle by fine‐tuning auxin asymmetric distribution in shoots.

## Introduction

Rice plant architecture is regarded as one of the main agronomical traits that influence planting density in the field and thus contribute grain yield (Khush, 2003). Rice is a multi‐tiller crop, and its plant architecture is determined by tiller angle, tiller number, plant height, leaf inclination and panicle architecture as well (Wang and Li, 2008). Tiller angle is defined as the angle between the main culm and its side tillers, and it is one of the main target traits being selected in breeding for achieving ideal plant architecture to improve rice yield. Therefore, understanding the mechanism controlling till angle will not only elucidate the process of varieties artificial selection but also provide a way of improving grain production in rice.

With the implementation of rice functional genomics project, quite a few genes have been characterized involving the control of tiller angle. *LAZY1* (*LA1*) is the first identified gene that controls rice tiller angle. Mutation in *LA1* alters the endogenous IAA distribution in shoots, leading to the tiller‐spreading phenotype in rice (Li *et al.*, 2007; Yoshihara and Iino, 2007). HSFA2D acts an upstream regulator of *LA1* to initiate the asymmetric auxin distribution and further influences asymmetric expression of *WOX6* and *WOX11* to specify tiller angle (Zhang *et al.*, 2018). Recent investigation showed that OsBRXL4 regulates shoot gravitropism and rice tiller angle through affecting LAZY1 nuclear localization (Li *et al.*, 2019). It was reported that strigolactones (SLs) biosynthetic or signalling mutants could rescue the spreading phenotype of *la1*, indicating that SLs also participate in the tiller angle regulation by inhibiting auxin biosynthesis (Sang *et al.*, 2014). The dominant *PAY1* could optimize tiller angle via affecting auxin transport activity and distribution in rice (Zhao *et al.*, 2015). *OsLIC* functions as a negative regulator for optimal tiller angle in rice through mediating the brassinosteroids (BRs) response (Wang *et al.*, 2008). *LPA1*, encoding a plant‐specific INDETERMINATE DOMAIN protein, controls tiller angle by regulating the sedimentation rate of amyloplasts (Wu *et al.*, 2013). Additionally, several quantitative trait loci (QTLs) affecting tiller angle have been characterized (Dong *et al.*, 2016; He *et al.*, 2017; Qian *et al.*, 2001; Yu *et al.*, 2007). Mutation in the major QTL *Tiller Angle Control1* (*TAC1*) leads to a compact tiller in *japonica* rice compared with relatively wider tiller angle in *indica* rice (Yu *et al.*, 2007). Another QTL *TAC3*, together with *TAC1* and *D2,* greatly controls tiller angle in rice cultivars (Dong *et al.*, 2016). Besides, tiller angle is also associated with rice domestication. The artificial selection of an amino acid substitution in the PROG1 protein during domestication resulted in the transition from the prostrate tillers of wild rice to the erect tillers of cultivated rice (Jin *et al.*, 2008; Tan *et al.*, 2008). *TIG1* encoding a TCP transcription factor also contribute to the transition from inclined tiller growth in wild rice to erect tiller growth during rice domestication (Zhang *et al.*, 2019).

MicroRNAs (miRNAs), a type of small (~21 nucleotides) non‐coding RNAs, play a crucial role in negative regulation of gene expression by binding to target mRNAs for cleavage and (or) translation repression at the post‐transcriptional level in both plants and animals (Bartel, 2009; Carrington and Ambros, 2003). Auxin response factors (ARFs) are transcription factors that bind specifically to TGTCTC‐containing auxin‐responsive elements (AuxREs) found in the promoters of early auxin response genes and mediate the signal transduction of auxin, and finally regulate plant growth and development (Chandler, 2016; Li *et al.*, 2016; Wang *et al.*, 2007). In plants, several *ARF* genes have been identified as the cleavage targets of miR390, miR160 and miR167. In *Arabidopsis*, *AtARF2*, *AtARF3* and *AtARF4* are the targets of miR390‐derived trans‐acting small interfering RNAs (ta‐siRNA) (Fahlgren *et al.*, 2006; Williams *et al.*, 2005). In *Arabidopsis*, miR160 and its targets *ARF10*, *ARF16* and *ARF17* have been suggested to function in growth and developmental processes. miR160 regulates the expression of *ARF10* and *ARF16* and determines the root cap formation (Wang *et al.*, 2005). miR160‐directed *ARF17* regulation is essential for proper development via modulating expression of early auxin response genes (Mallory *et al.*, 2005). Repression of *ARF10* by miR160 plays a critical role in seed germination and post‐embryonic developmental programmes (Liu *et al.*, 2007). Furthermore, miR160‐controlled *ARF10* is essential for seed germination through stimulation of both auxin and ABA signalling (Liu *et al.*, 2013). *ARF6* and *ARF8* were identified as the cleavage targets of miR167 in *Arabidopsis* (Ru *et al.*, 2006; Wu *et al.*, 2006; Yao *et al.*, 2019; Zheng *et al.*, 2019). Previous investigation showed that miR167‐directed *ARF6*/*ARF8* expression is essential for reproductive development in *Arabidopsis* (Ru *et al.*, 2006; Wu *et al.*, 2006). Recent studies suggested that miR167‐mediated negative regulation of *ARF6* and *ARF8* is necessary for anther dehiscence and ovule development (Zheng *et al.*, 2019). Yao *et al. *(2019) reported that *MIR167a* acts as a maternal gene and functions largely through *ARF6* and *ARF8* in controlling of embryonic and seed development. Blue light could increase miR167 expression and then resulted in the enhanced transcription of *ARF4* and *ARF8* (Pashkovskiy *et al.*, 2016). Interestingly, *ARF17* (a target of miR160) is a negative regulator, and *ARF6* and *ARF8* (targets of miR167) are positive regulators of adventitious rooting, suggesting that miR160 and miR167 commonly control phenotypic plasticity of adventitious root initiation in *Arabidopsis* (Gutierrez *et al.*, 2009).

MiR167 targeted the transcription factor *ARF* genes had been reported to be conserved among other plant species, including rice (Liu *et al.*, 2012a), soybean (Wang *et al.*, 2015), tomato (Liu *et al.*, 2014), *Ipomoea nil* (Glazinska *et al.*, 2014), *Camelina sativa* (Na *et al.*, 2019) and so on. A total of 25 putative *OsARFs* have been identified in of rice (Guilfoyle and Hagen, 2007; Li *et al.*, 2016; Wang *et al.*, 2007). Four *OsARFs* (*OsARF6*, *OsARF12*, *OsARF17* and *OsARF25*) were predicted to be the target of OsmiR167 (Li *et al.*, 2010; Liu *et al.*, 2012a). Yang *et al. *(2006) reported that OsmiR167‐*OsARF8*‐*GH3* was response to exogenous auxin in cultured rice cells. OsmiR167‐directed *OsARF12* expression regulates root elongation and affects iron accumulation in rice (Qi *et al.*, 2012). In order to determine the biological function of OsmiR167, we generated *OsMIR167a*‐overexpressing lines in rice. *OsARF12*, *OsARF17* and *OsARF25* were examined as the cleavage targets of OsmiR167a. Both overexpressing *OsMIR167a* lines and *osarf12/osarf17* and *osarf12/osarf25* mutants showed larger tiller angle phenotype and disrupted auxin distribution in axillary buds. Our results indicate that OsmiR167a regulates the pattern of *OsARF12*, *OsARF17* and *OsARF25* expression, which is vital for modulation of tiller angle in rice.

## Results

### Overexpressing *OsMIR167a* resulted in larger tiller angle in rice

According to the annotation of miRNA database (miRBase: http://www.mirbase.org/index.shtml) (Kozomara and Griffiths‐Jones, 2011, 2014), there are ten *OsMIR167* (*OsMIR167a* to *OsMIR167j*) genes in rice genome. Although *OsMIR167* genes harbour different stem‐loop precursors, they generate the similar mature sequences of OsmiR167. There is a single nucleotide difference in the 3′ end between OsmiR167a‐c and OsmiR167d‐j, and OsmiR167a‐c show the same sequence with *Arabidopsis* AtmiR167a‐b (Table S1). In order to elucidate the function of miR167 in rice, we took *OsMIR167a* as the representative to generate its overexpressing plants (designated as *MIR167a‐OE*).

**Figure 1:**
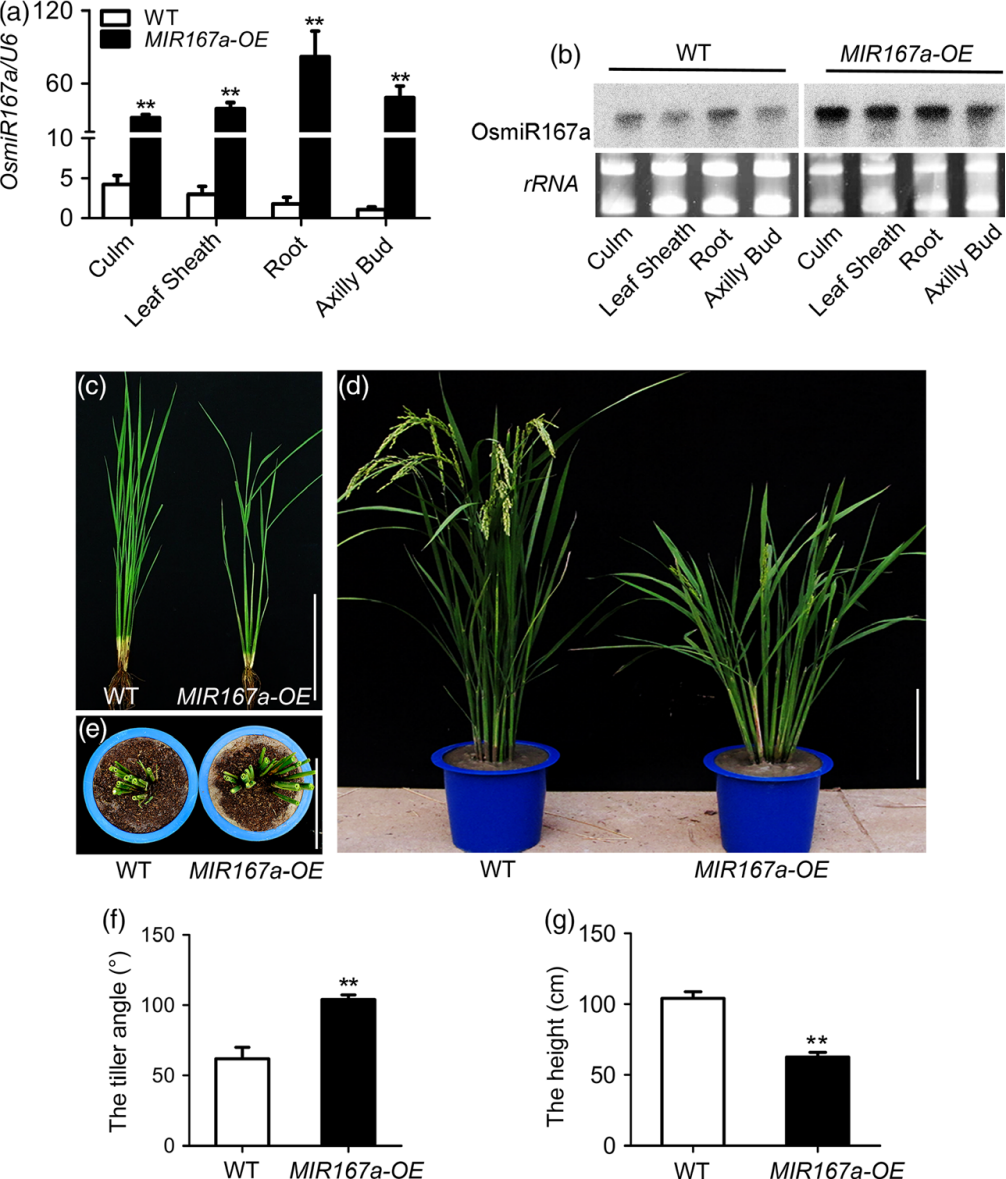
Phenotypic analysis of *MIR167a‐OE* lines. (a, b) Expression analysis of miR167a in the culm, leaf sheath, root and axillary bud by quantitative real‐time RT‐PCR (qRT‐PCR) (a) and small RNA gel blot (b). *U6* was used as a control. (c, d) Plant morphology of WT (ZH11) and *MIR167a‐OE* lines at tillering (c) and heading (d) stages. (e) Vertical observation of WT and *MIR167a‐OE* transgenic lines. (f, g) Comparison of the tiller angle (f) and plant height (g) between WT and *MIR167a‐OE* lines. Data are shown as means ± SE (*n* = 10). Bars = 20 cm. Significant at **P < 0.01.

We first examined the expression pattern of *OsMIR167a* in different organs at vegetative stage. Quantitative real‐time RT‐PCR (qRT‐PCR) showed that pre‐OsmiR167a was detected in the examined organs including culm, leaf sheath, root and axillary bud (Figure 1a). The expression pattern of OsmiR167a was further confirmed by RNA gel blot analysis (Figure 1b). In the *MIR167a‐OE* lines, pre‐OsmiR167a and OsmiR167a had higher expression levels in those organs compared to that in wild‐type plants (WT) (Figure 1a,b). Then, we characterized the morphological differences between *MIR167a‐OE* and WT. At tillering stage, *MIR167a‐OE* plants showed clearly larger tiller angle compared with WT (Figure 1c). After heading, tiller angle was increased dramatically in *MIR167a‐OE* plants (Figure 1d,e). Compared to WT, *MIR167a‐OE* plants showed obvious semi‐dwarf and low fertility (Figure 1c–g). The maximum tiller angle of among *MIR167a‐OE* plants was 110°, approximately 40° wider than that in WT (Figure 1e,f). The plant height of *MIR167a‐OE* was reduced about 40% of the WT (Figure 1g). Our results suggest that overexpression of *MIR167a* results in larger tiller angle in rice.

### *OsARF12*, *OsARF17* and *OsARF25* are the promising target genes of OsmiR167a

**Figure 2:**
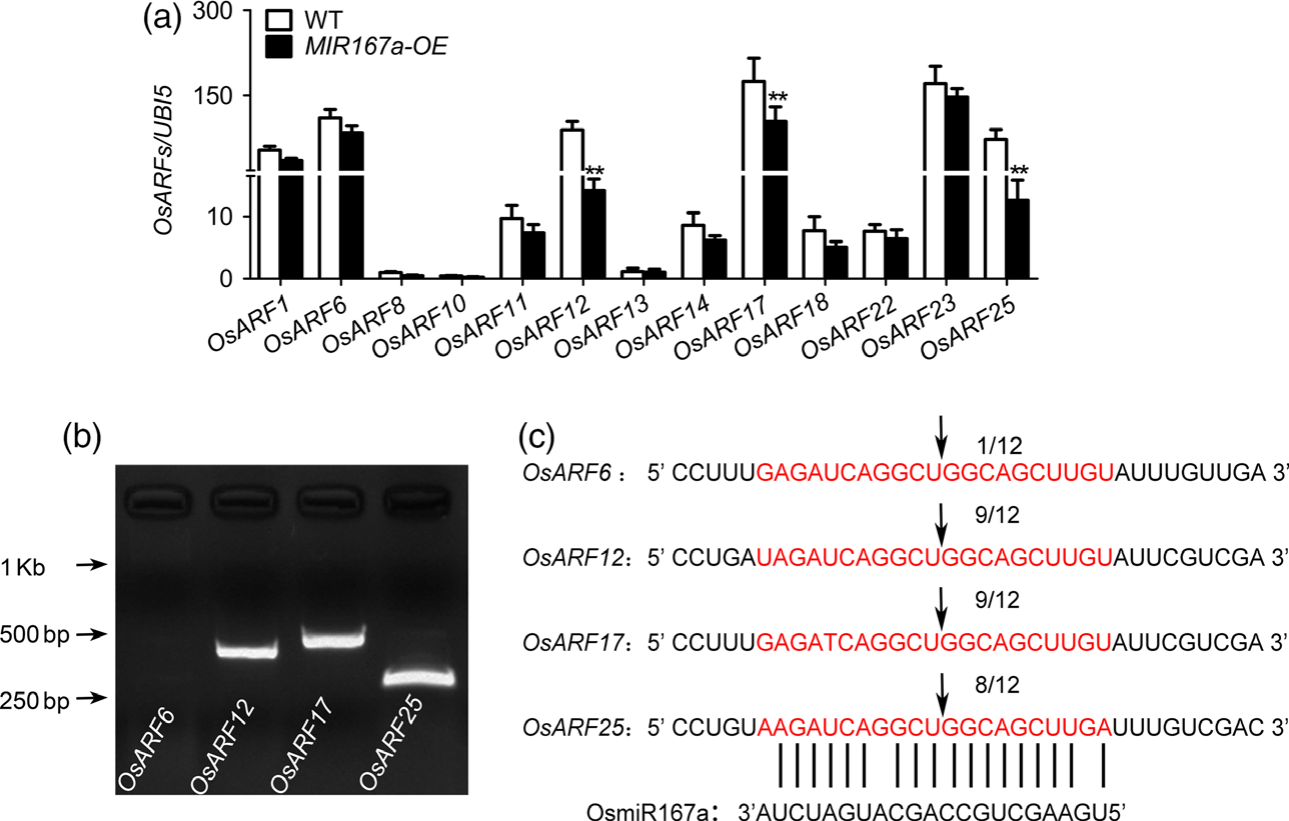
Mapping of miR167 target cleavage sites in *OsARF12*, *OsARF17* and *OsARF25*. (a) Expression pattern of predicted target genes in WT and *MIR167a‐OE* plants. *Ubiquitin 5* was used as a control. Data are presented as means ± SE (*n* = 3). Significant at **P < 0.01. (b) PCR products of *OsARF6*, *OsARF12*, *OsARF17* and *OsARF25* by RLM‐5'RACE was showed by gel electrophoresis. (c) The sequencing results of 12 clones from the 5′ RACE cleavage products of *OsARF6, OsARF12, OsARF17* and *OsARF25*. The vertical lines represent the nucleotides that base‐pair with miR167a. The arrow points to the OsmiR167‐directed cleavage site at the *OsARFs* transcript. The numbers above the sequence indicate frequency of 5′RACE clones corresponding to each site.

There are 25 *OsARFs* in rice. Previous investigations predicted that *OsARF6, OsARF12*, *OsARF17* and *OsARF25* were the putative target genes of OsmiR167a (Liu *et al.*, 2012a; Zhang, 2005). We further verified the predicted miRNA targets by CSRDB (Cereal small RNAs Database; http://sundarlab.ucdavis.edu/smrnas/) (Table S2). RT‐PCR analysis of *OsARF* genes expressed in axillary buds suggested that the expression levels of *OsARF12, OsARF17* and *OsARF25* were significantly reduced in *MIR167a‐OE* plants, whereas *OsARF6* and other *OsARFs* (*OsARF1, OsARF8, OsARF10‐11, OsARF13‐14, OsARF18, OsARF22*) performed no significantly decreased expression (Figure 2a). We further examined the OsmiR167a‐directed cleavage sites of *OsARFs* using a 5′ RNA ligase‐mediated (RLM) rapid amplification of cDNA ends (RACE) assay. The cleavage products of *OsARF12, OsARF17* and *OsARF25* mRNA fragments were successfully detected in *MIR167a‐OE* plants, except for that of *OsARF6* (Figure 2b). We randomly chose 12 clones from the cleavage products for sequencing, and more than 8 clones showed that the transcripts of *OsARF12, OsARF17* or *OsARF25* were cleaved at the OsmiR167a target site (Figure 2c). Thus, *OsARF12*, *OsARF17* and *OsARF25* might be the target genes of OsmiR167a in rice.

### OsmiR167a‐*OsARFs* modules regulate tiller angle in rice

**Figure 3:**
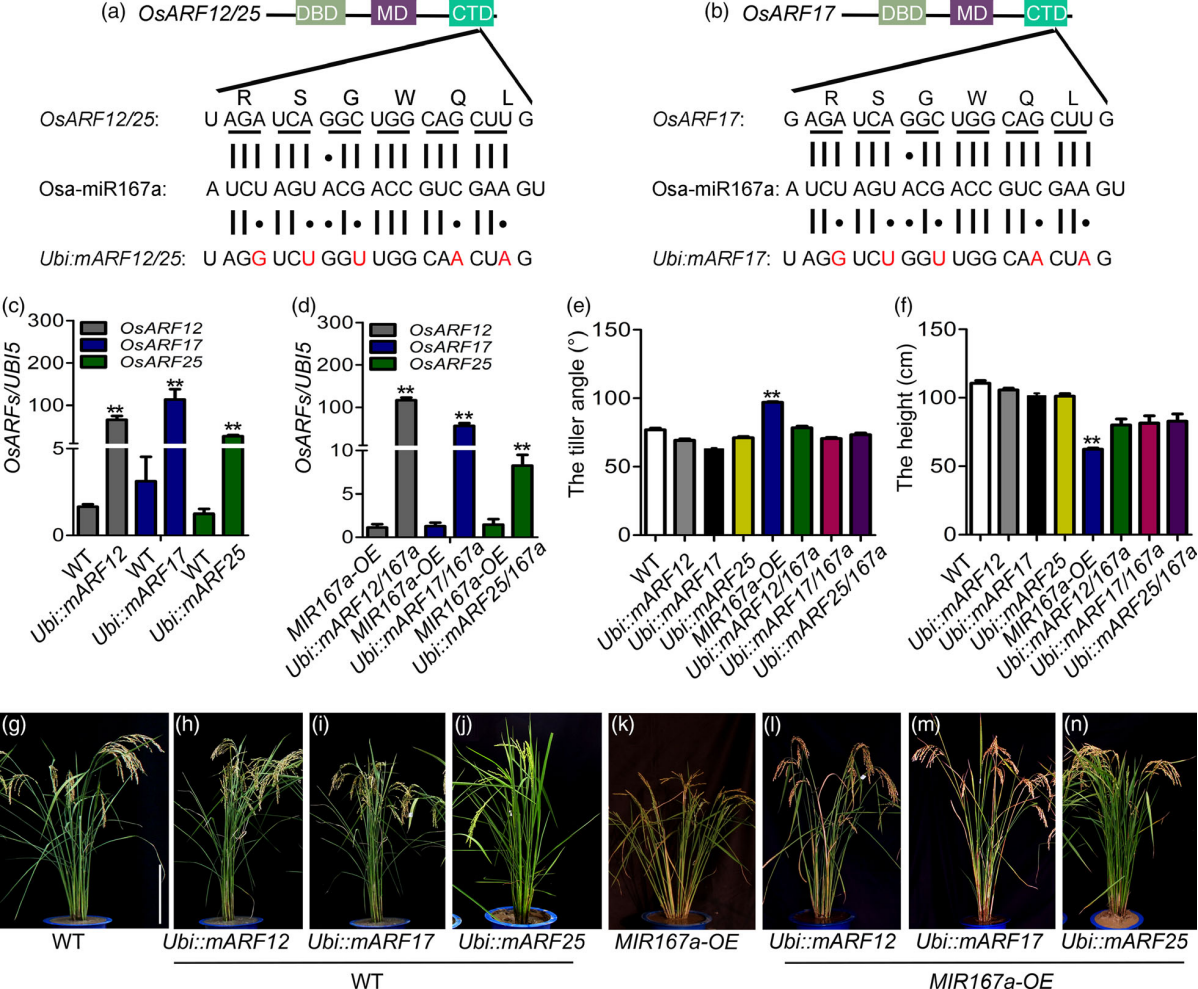
Generation of transgenic rice plants expressing an OsmiR167‐resistant version of *OsARFs* (*mOsARFs*). (a, b) Domain structure of the OsARF12, OsARF17 and OsARF25 protein. The B3 DNA binding domain (DBD), middle region (MR) and the C‐terminal dimerization domain (CTD) are indicated. The OsmiR167 complementary (or target) sequence in the *OsARFs* mRNA and the corresponding region of amino acid sequence (RSGWQL) are shown. The silent mutations were created in *mOsARFs* by introducing silent mutations. The vertical lines represent the nucleotides that base pair with miR167a and black dots show the mismatched nucleotide. (c, d) Expression levels of uncleaved *OsARF12*, *OsARF17* and *OsARF25* by qRT‐PCR in transgenic lines. (e, f) Comparison of the tiller angle (e) and plant height (f) between WT and transgenic lines. (g–n) Phenotype of *Ubi::mOsARFs* and *Ubi::mOsARFs*/*167a* plants. Bar = 20 cm. Significant at **P < 0.01.

To confirm that *OsARF12*, *OsARF17* and *OsARF25* are the target genes of OsmiR167a, we generated a series of transgenic plants overexpressing OsmiR167a‐resistant version of individual *OsARFs* (resulting plants named *Ubi::mARF12*, *Ubi::mARF17* and *Ubi::mARF25*) in Zhonghua 11 (WT) and *MIR167a‐OE* background, respectively. Based on the conserved carboxy‐terminal dimerization domain (CTD) of *OsARFs,* which contained the OsmiR167a‐directed cleavage site, five synonymous mutations were introduced into the transgenic constructs (Figure 3a,b) without any amino acid changes. qRT‐PCR analysis confirmed the positive transgenic lines had overexpression of un‐cleaved version of *OsARF12*, *OsARF17* and *OsARF25* in WT (Figure 3c) and *MIR167a‐OE* (Figure 3d) background, respectively. Next, we characterized the morphological changes of the *Ubi::mARF12*, *Ubi::mARF17* and *Ubi::mARF25* transgenic plants compared to WT or *MIR167a‐OE* (Figure 3e–n). In WT background, transgenic plants with *Ubi::mARF12*, *Ubi::mARF17* and *Ubi::mARF25* showed slight reduced tiller angle compared with WT (Figure 3e,g–j). No remarkable plant height changes were observed between *OsARFs‐*resistant plants and WT (Figure 3f,g–j). However, in *MIR167a‐OE* background, overexpressing OsmiR167a‐resistant version of individual *OsARF12, OsARF17* or *OsARF25* resulted in obviously decreased tiller angle compared with *MIR167a‐OE* plants (Figure 3e,k–n). Meanwhile, the semi‐dwarf of *MIR167a‐OE* plants was partially restored in *OsARFs‐*resistant plants (Figure 3f,k–n). These observations suggest expression of *mOsARF12, mOsARF17* or *mOsARF25* could partially rescue the abnormal plant architecture when *OsMIR167a* overexpressed*.*

Target *MIMICs* are known to inhibit miRNA activity (Franco‐Zorrilla *et al.*, 2007; Todesco *et al.*, 2010). Thus, we further verified the OsmiR167a‐*OsARFs* involved in tiller angle regulation by generation the *MIM167a* transgenic plants. Three‐nucleotide bulge (CAA) were introduced into the mature OsmiR167a, which replaced the OsmiR399 complementary motif in *OsIPS1* (Franco‐Zorrilla *et al.*, 2007) to engineer an artificial target mimicry construct of *MIM167a* (Figure S1a). In the *MIM167a* transgenic plants, the abundance of *OsARF12, OsARF17* or *OsARF25* were significantly increased relative to that in *MIR167a‐OE* plants, but not in WT background (Figure S1b). In WT background, overexpression of *MIM167a* has no effect on the tiller angle and plant height (Figure S1c,e,f). However, in *MIR167a‐OE* plants, the morphologies of larger tiller angle and semi‐dwarf were partially restored when overexpressing *MIM167a* (Figure S1d–f). Collectively, these results suggest that OsmiR167a modulates rice tiller angle by fine‐tuning the accumulation of *OsARFs,* mainly including *OsARF12, OsARF17* and *OsARF25*.

### *OsARF12*, *OsARF17* and *OsARF25* function redundantly to modulate tiller angle

**Figure 4:**
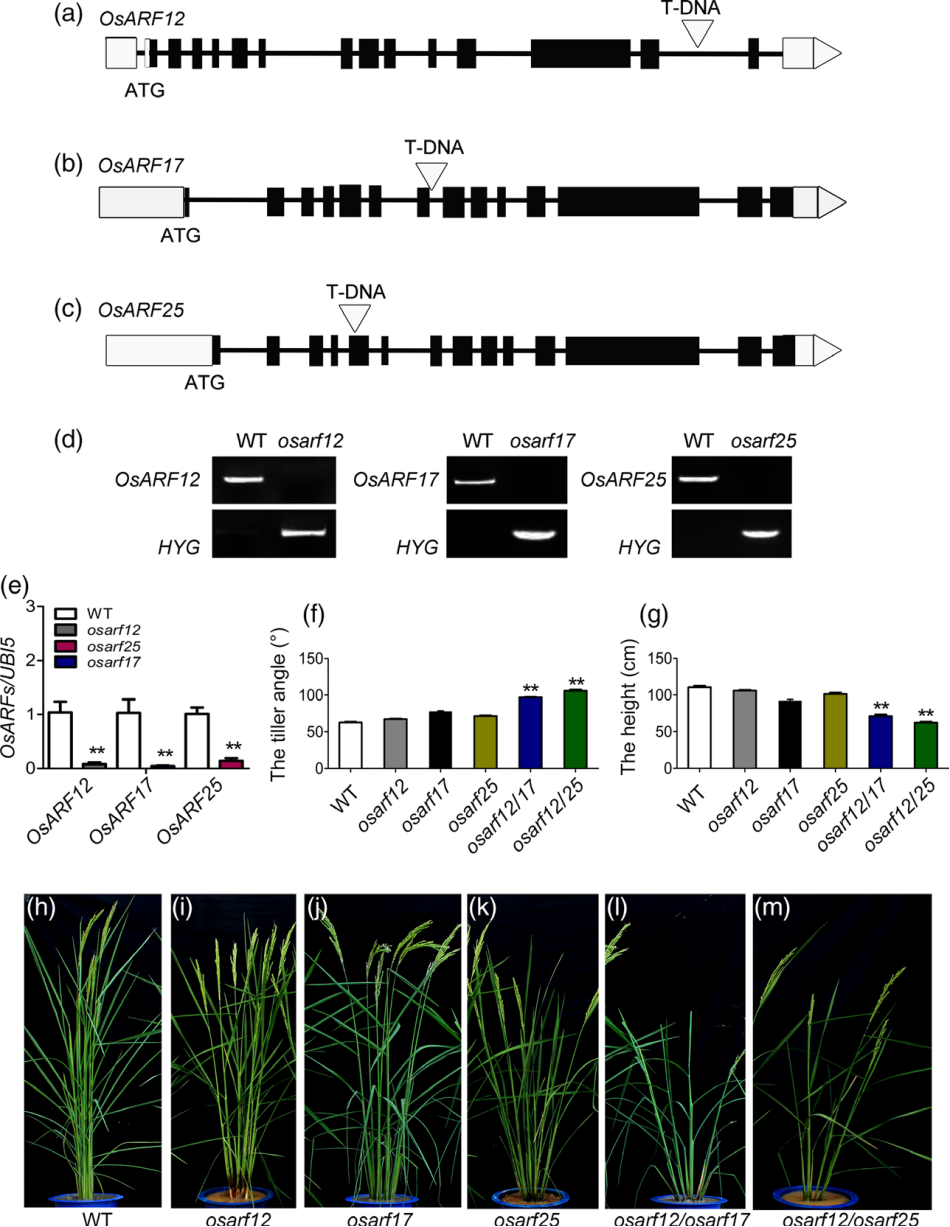
Identification of *OsARFs* mutants. (a–c) T‐DNA insertion sites in *osarf12*, *osarf17* and *osarf25*. Exons (filled boxes) and introns (lines between the filled boxes) are shown. The open triangle indicates T‐DNA insertion site. (d) PCR analysis to confirm the integration of T‐DNA in *OsARF* mutants; the upper band indicates the *OsARF* gene fragment and the lower band indicates the T‐DNA insertion fragment. (e) Expression analysis the level of *OsARF12*, *OsARF17* and *OsARF25* in WT and *osarf* mutants by qRT‐PCR. (f–g) Comparison of the tiller angle (f) and plant height (g) of WT and mutants. (h–m) Morphologies of WT and mutants. Bar = 20 cm. Significant at **P < 0.01.

To further confirm the role of *OsARF12*, *OsARF17* and *OsARF25* in tiller angle control, we collected their mutants and developed the double mutants to characterize the plant architecture. We obtained *osarf12* (03Z11JV18) and *osarf17* (03Z11BY76) mutants from our T‐DNA insertional mutant library in variety Zhonghua 11 (Wu *et al.*, 2003). Analysis of T‐DNA insertion sites in the mutants shows that it is located in the fourteenth intron of *OsARF12* (Figure 4a) and seventh intron of *OsARF17* (Figure 4b), respectively. For *osarf25* (PFG_2D‐11520) in variety Dongjing, T‐DNA insertion site in the fifth exon was characterized previously (Figure 4c; Qi *et al.*, 2012). PCR analysis confirmed that the T‐DNA inserted into individual *OsARF* genes, and their homozygous were obtained (Figure 4d). qRT‐PCR results indicated the transcripts of *OsARF12*, *OsARF17* and *OsARF25* were suppressed in its corresponding mutants (Figure 4e), suggesting that these genes were knockout in mutants. We further characterized the tiller angle and plant height of *osarf12*, *osarf17* and *osarf25* compare with WT (Figure 4f–m). At the heading stage, *osarf12* and *osarf17* showed slight loosen plant types (Figure 4f,h–j) with tiny increase tiller angle compared to WT, and no obvious changes on the plant height were observed (Figure 4g–j). However, *osarf25* showed obviously increased tiller angle and reduced plant height compared to WT (Figure 4f–k). The double mutants of *osarf12*/*osarf17* and *osarf12*/*osarf25* displayed remarkable phenotype defects with larger tiller angle and semi‐dwarf (Figure 4f–g,l–m). Thus, our results suggest that *OsARF12*, *OsARF17* and *OsARF25* function redundantly to modulate tiller angle.

### Auxin up‐regulated expression of *OsARF12*, *OsARF17* and *OsARF25*

To test whether the expression of *OsARF12*, *OsARF17* and *OsARF25* was affected by phytohormones, we treated 7‐day seedlings with various hormones for 2 h. qRT‐PCR results showed that the expression of *OsARF12*, *OsARF17* and *OsARF25* were significantly induced by IAA treatment, but not by the other hormones (Figure S2a–c). Moreover, two other auxin compounds, IBA (indole‐3‐butyric acid) and NAA (naphthylacetic acid) also can induce the expression of *OsARF12*, *OsARF17* and *OsARF25*, but with a less extent (Figure S2a–c). We further used tryptophan (a structurally similar molecule to IAA) to treat the seedlings, the expression of *OsARF12*, *OsARF17* and *OsARF25* remained unchanged, indicating tryptophan has no hormonal activity (Figure S2a–c). Next, we examined the time‐course response of *OsARF12*, *OsARF17* and *OsARF25* to 10 μM IAA treatment. The transcripts of *OsARF12*, *OsARF17* and *OsARF25* showed remarkable increase up to 60 min of IAA application (Figure S2d). However, the transcript levels of *OsARF12*, *OsARF17* and *OsARF25* maintained almost the same under different concentration of auxin (Figure S2e). Taken together, our results suggest that *OsARF12*, *OsARF17* and *OsARF25* might be involved in auxin response to modulate tiller angle in rice.

### OsmiR167a‐*OsARFs* modules affect polar auxin transport

**Figure 5:**
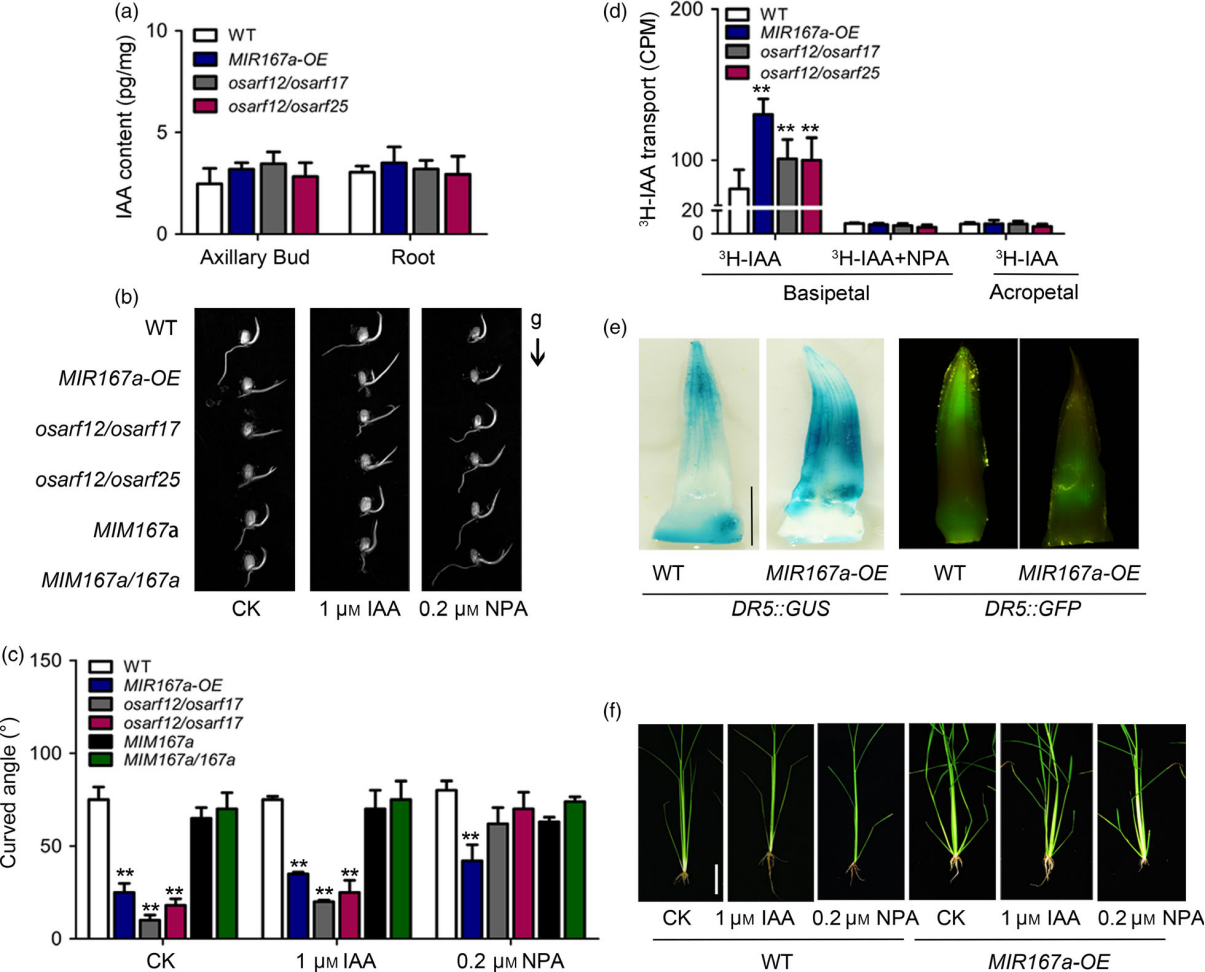
Altered auxin distribution and transport in *MIR167a* transgenic plant. (a) Quantification of auxin content in WT, *MIR167a‐OE* and *OsARF* mutant plants. (b) Three‐day‐old seedlings of the WT, *MIR167a‐OE*, ARF double mutants and *MIM167a* transgenic plants grown in dark after a 24‐h gravistimulation. The arrow indicates the direction of gravity (g). (c) Statistical data of ultimate curved angle of WT, *MIR167a‐OE*, *OsARF* double mutants and *MIM167a* transgenic lines under IAA or NPA treatment. Data are shown as means ± SE (*n* = 10). (d) Measurement of auxin transport ability in WT, *MIR167a‐OE* and *OsARF* mutant plants using coleoptile as materials. Significant at **P < 0.01. (e) Comparison of *DR5* promoter activity in axillary bud of WT and *MIR167a‐OE* plants. Bar = 0.5 cm. (f) Morphology of *MIR167a‐OE* plants under treatment of 1 μM IAA and 0.2 μM NPA treatments for 40 days. Bar = 5 cm.

Considering *OsARF12*, *OsARF17* and *OsARF25* may control rice tiller angle via auxin signalling, we carried out to examine whether OsmiR167a‐*OsARF* module is involved in sensing gravistimulation and polar auxin transport (PAT). We first examined the free IAA concentration in axillary buds and roots among *MIR167a‐OE, osarf12/osarf17, osarf12/osarf25* and WT plants. Compared to WT, the content of free IAA is almost consistent in *MIR167a‐OE, osarf12/osarf17* or *osarf12/osarf25* (Figure 5a), suggesting that OsmiR167a‐*OsARFs* may not affect free IAA accumulation in rice. However, gravitropism analysis of the WT and mutant seedlings showed that the coleoptiles of *MIR167a‐OE, osarf12/osarf17* and *osarf12/osarf25* could not grow upright completely. Correspondingly, *MIM167a*, *MIM167a*/*167a* and WT showed the normal gravity response, with upright coleoptiles (Figure 5b). When NPA (N‐1‐naphthylphthalamic acid, an auxin transport inhibitor) was added to the medium, the curved angle of coleoptiles of *MIR167a‐OE, osarf12/osarf17* and *osarf12/osarf25* were recurred, almost similar to that in WT (Figure 5b,c). However, this phenomenon of recurred curved angle was not observed when using IAA instead of NAA (Figure 5b,c). Thus, the gravitropism response requires the asymmetric distribution of auxin. Our result suggests that OsmiR167a‐*OsARFs* might affect gravity response by participating in auxin transport in rice.

Next, we compared the auxin transport in etiolated coleoptiles between WT and mutant lines. Compared to WT, the basipetal transport of ^3^H‐IAA was significantly increased in *MIR167a‐OE, osarf12/osarf17* and *osarf12/osarf25*, whereas the acropetal PAT of ^3^H‐IAA displayed no differences between WT and mutant lines (Figure 5d). When treated with NPA (N‐1‐naphthylphthalamic acid, an auxin transport inhibitor), the difference of basipetal PAT between WT and mutant lines was disappeared (Figure 5d). We further examined endogenous IAA distribution in the axillary buds by introducing the auxin reporter *DR5::GUS* and *DR5::GFP* into *MIR167a‐OE* background, respectively. In the axillary buds, the signal of GUS/GFP was mainly detected at the apex area in WT, whereas in *MIR167a‐OE* lines, the GUS/GFP signal moved to the middle part of axillary buds and expanded to a much broader area (Figure 5e), indicating that the enhanced basipetal PAT affected endogenous IAA distribution in axillary bud of *MIR167a‐OE* lines. Furthermore, the tiller angle was decreased in *MIR167a‐OE* when treating its seedlings with NPA (Figure 5f). Our results suggest that OsmiR167a‐*OsARFs* modules control rice tiller angle by affecting polar auxin transport in axillary buds.

### OsmiR167a‐*OsARFs* modules regulate tiller angle through mediating asymmetric auxin distribution in shoots

**Figure 6:**
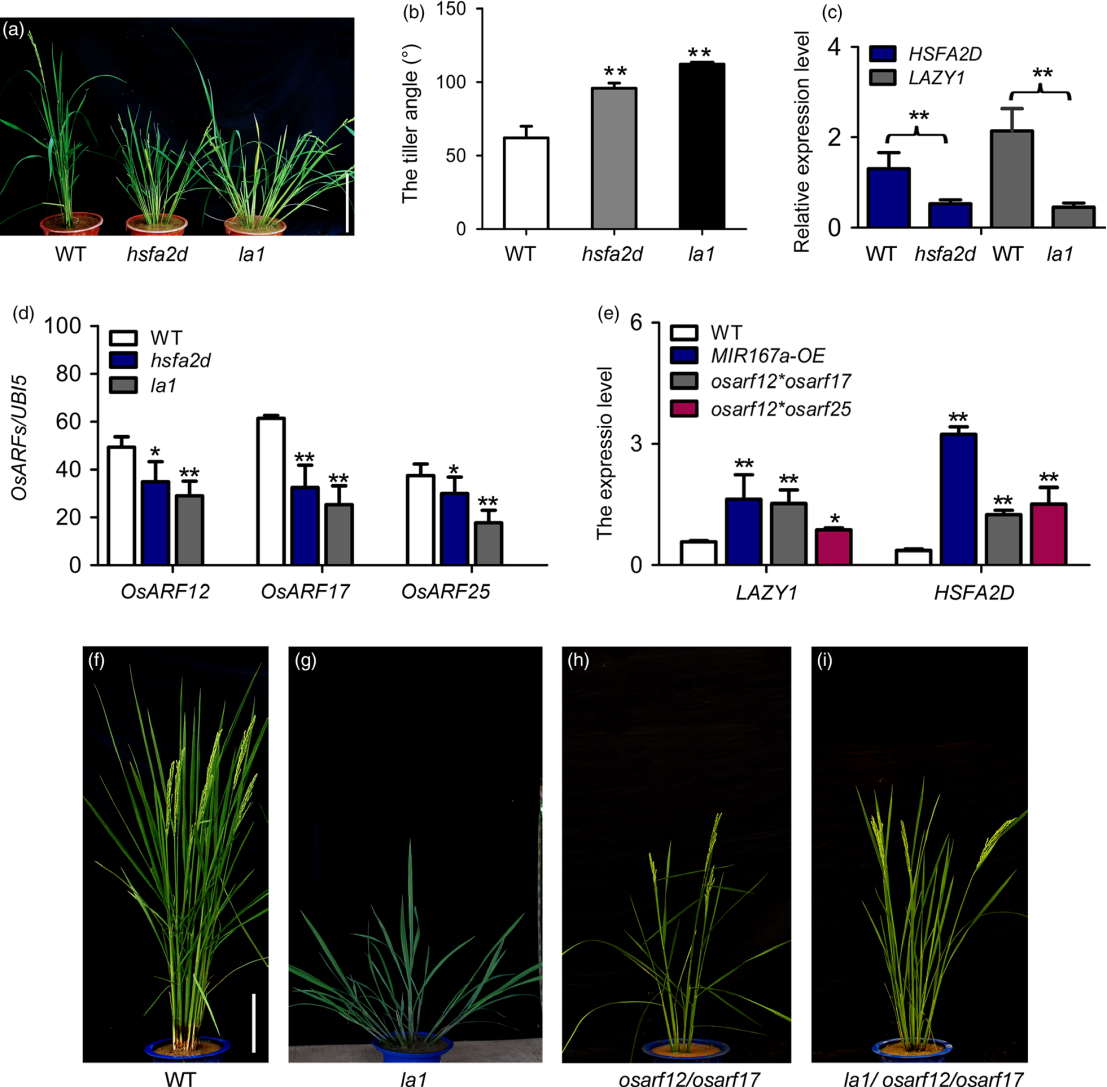
*OsARF12/17/25* and *LA1* affect auxin distribution in controlling tiller angle. (a, b) Morphology of WT, *hsfa2d* and *la1* mutants. Bar = 15 cm. (c) Transcript levels of *HSFA2D* and *LAZY1* in WT and mutants determined by qRT‐PCR. (d) Expression level of *OsARF12*, *OsARF17* and *OsARF25* in WT, *hsfa2d* and *la1* mutants. (e) Expression level of *HSFA2D* and *LAZY1* in WT and *OsARF* double mutants. Significant at *P < 0.05, **P < 0.01. (f–i) Phenotypes of WT, *la1*, *osarf12/osarf17* and *la1/osarf12/osarf17* plants. Bars = 20 cm.

Previous studies described a regulatory pathway of rice tiller angle by *HSFA2D*‐*LAZY1* (*LA1*)–mediated auxin asymmetric distribution (Zhang *et al.*, 2018). A knockout mutant *hsfa2d* (03Z11CE69) showing remarkable larger tiller angle has been identified from our T‐DNA insertional mutant library (http://rmd.ncpgr.cn/; Zhang *et al.*, 2018). In order to investigate whether OsmiR167a‐*OsARF* module is involved in the *LAZY1*‐mediated asymmetric auxin distribution pathway, we obtained *lazy1* mutant (03Z11UL88) from our T‐DNA insertional mutant library by mapping‐based cloning approach (Figure S3). We identified a 69 bp deletion in the 2^nd^ exon of *LAZY1* (*LA1*), which resulted in large tiller angle in *la1* (Figure S3; Table S3). Both *hsfa2d* and *la1* displayed an obvious larger tiller angle and semi‐dwarf phenotypes (Figure 6a,b). RT‐PCR results showed that the transcripts of *HSFA2D* and *LA1* were significantly suppressed (Figure 6c), indicating that both *HSFA2D* and *LA1* are loss of function in their corresponding mutants. Compared to that of WT, the expression levels of *OsARF12*, *OsARF17* and *OsARF25* were significantly reduced in *hsfa2d* or *la1* in axillary shoots using qRT‐PCR analysis (Figure 6d). However, in the *MIR167a‐OE, osarf12/osarf17* or *osarf12/osarf25* backgrounds, the expression of *HSFA2D* and *LA1* were obviously up‐regulated (Figure 6e), indicating that a feedback regulation network might exist between HSFA2D‐*LA1* and OsmiR167a‐*OsARFs* modules. Of course, further analysis of the expression levels of *OsMIR167a* and *OsARF12/1/7/25* in the overexpression of *HSFA2D* or *LA1* plants will be needed to confirm this speculation. Compared to WT, the *la1* showed more larger tiller angle than that of *osarf12/osarf17* double mutant (Figure 6f–h). The *la1*/*osarf12/osarf17* triple mutant exhibited an intermediate larger tiller angle between that of *la1* and *osarf12/osarf17* (Figure 6g–i), suggesting that OsmiR167a‐*OsARFs* modules might also be involved in the asymmetric auxin distribution in controlling tiller angle.

Zhang *et al. *(2018) has reported that *HSFA2D‐LA1* induces the asymmetric distribution of auxin, and then auxin triggers the asymmetric expression of *WOX6* and *WOX11* in shoots mediating the control of rice tiller angle. Thus, we further examined the expression patterns of *WOX6* and *WOX11* in seedling shoots upon gravistimulation under light for 6h in *MIR167a‐OE*, *osarf12/osarf17* and *osarf12/osarf25* plants. Similar to previous investigation (Zhang *et al.*, 2018), the expression of *WOX6* and *WOX11* indeed performed asymmetric expression patterns with highly expressed in the lower side of shoot bases (Figure S4). However, in the *MIR167a‐OE, osarf12/osarf17* or *osarf12/osarf25* plants, the extent of the induction of *WOX6* and *WOX11* expression in the lower side of shoot bases was significantly reduced, compared to that in WT (Figure S4). Considering OsmiR167a‐*OsARFs* modules affect polar auxin transport in axillary buds (Figure 5d,e), these results suggest that OsmiR167a‐*OsARFs* modules might be involved in the asymmetric distribution of auxin in rice shoots and then resulted in the asymmetric expression of *WOX6* and *WOX11* to modulate tiller angle in rice.

## Discussion

### *OsARF12, OsARF17* and *OsARF25* are required to regulate tiller angle

There are 25 *OsARF* genes in rice genome (Guilfoyle and Hagen, 2007; Wang *et al.*, 2007). All of the OsARF proteins contain a highly conserved N‐terminal domain for DNA binding, a central region, and a C‐terminal domain for protein‐protein interaction (Shen *et al.*, 2010; Wang *et al.*, 2007). Although a few *OsARF* genes have been functional characterized, the functions for most of them remain unknown. In this study, we identified OsmiR167a‐*OsARF12/17/25* as a key regulatory module of tiller angle control, which affects plant architecture in rice.

*OsARF1* is the first ARF gene that regards as an early auxin‐responsive gene in rice (Waller *et al.*, 2002). The morphological defects of antisense‐*OsARF1* plants suggested that *OsARF1* is essential for vegetative growth and seed development (Attia *et al.*, 2009). *OsARF3* mediates the auxin‐cytokinin cross‐talk involving de novo shoot regeneration (Cheng *et al.*, 2013). *OsARF12* functions in phosphate homeostasis and required for primary root elongation (Qi *et al.*, 2012; Wang *et al.*, 2014). *OsARF16* is required for the auxin and cytokinin response and is involved in phosphate transport and signalling (Shen *et al.*, 2014). *OsARF17* and *OsARF19* control rice leaf inclination through regulating both auxin and BR signalling or cross‐talk between them (Chen *et al.*, 2018; Zhang *et al.*, 2015). OsARF23 and OsARF24 control cell growth and morphogenesis through heterodimerization to reduce the expression of RMD in rice (Li *et al.*, 2014). In rice, 25 ARF protein sequences were classified into three clusters according to the phylogenetic tree (Wang *et al.*, 2007). OsARF12, OsARF17 and OsARF25 belonged to the same subgroup and were phylogenetically closest to each other, suggesting that they might perform the similar biological function. In this study, we obtained the T‐DNA insertion mutant lines for *OsARF12*, *OsARF17* and *OsARF25* (Figure 4). Their single mutant plant showed tiny increase tiller angle compared to WT, but the double mutants of *osarf12*/*osarf17* and *osarf12*/*osarf25* displayed remarkable larger tiller angle (Figure 4h–m). Thus, our studies demonstrate that *OsARF12*, *OsARF17* and *OsARF25* function redundantly to modulate tiller angle.

### miR167 target *ARF* gene*s* are conserved in plants

Sequence similarities of mature miRNAs from various species indicated that miR167 is conserved throughout the plant kingdom (Li *et al.*, 2010; Luo *et al.*, 2013). Comparison of mature miR167s between *Arabidopsis* and rice showed the identical sequence (Table S1). Although some non‐conserved transcripts besides *ARF* gene family were predicted as targets for miR167 (Li *et al.*, 2010), many *ARF* genes were experimentally validated as the miR167 targets among different species. In *Arabidopsis*, miR167 targets *AtARF6* and *AtARF8*, and regulates some reproductive development process, such as anther dehiscence, ovule development, embryonic and seed development (Ru *et al.*, 2006; Wu *et al.*, 2006; Yao *et al.*, 2019; Zheng *et al.*, 2019). In rice, four *OsARFs* (*OsARF6*, *OsARF12*, *OsARF17* and *OsARF25*) were predicted as the target of OsmiR167 (Liu *et al.*, 2012a), and they belong to the same subgroup with *AtARF6* and *AtARF8* in *Arabidopsis* by phylogenetic analysis (Wang *et al.*, 2007). In this study, we characterized the function of *OsARF12*/*17*/*25*, orthologues of *Arabidopsis AtARF6/8*. Overexpression of OsmiR167a or knock out of its targets, the *OsARF* genes (*OsARF12*, *OsARF17* and *OsARF25*), has led to larger tiller angle in rice (Figures 1d, 4). Therefore, our data demonstrate that rice OsmiR167a target *OsARF12*/*17*/*25* control plant architecture via affecting tiller angle. In soybean, *GmARF8a* and *GmARF8b* are the homologous genes of *Arabidopsis AtARF8*. The miR167‐GmARF8 module is required for soybean nodulation and lateral root development (Wang *et al.*, 2015). The tomato *SlARF6* and *SlARF6* genes, as the orthologous *AtARF6* and *AtARF8* genes in *Arabidopsis*, are also the targets of miR167 (Liu *et al.*, 2014). *SlARF6* and *SlARF6* have conserved roles in controlling growth and development of vegetative and flower organs in tomato (Liu *et al.*, 2014). Recently, Na *et al. *(2019) reported that overexpression of miR167 targeted the *CsARF8* in *Camelina* causes decreased content of α‐linolenic acid and increased seed size. Collectively, miR167 target ARF genes are conserved, but play diverse roles in plant growth and development.

Besides *OsARF12*, *OsARF17* and OsARF25, *OsARF6* also reported as the target of OsmiR167 (Liu *et al.*, 2012a). However, the *OsARF6* mRNA level was not reduced significantly in *MIR167a‐OE* plants in this study (Figure 2a). On the other hand, the cleavage products of *OsARF6* mRNA fragments were rarely detected in *MIR167a‐OE* plants by RLM‐5'RACE (Figure 2b,c). It is possible that miR167‐mediated repression of *OsARF6* function is at the level of translation. Whether *OsARF6* also functions in rice tiller angle control, we need to develop its mutant for in‐depth dissection of *OsARF6* functions.

### Asymmetric auxin distribution affects tiller angle in rice

Rice tiller angle is a complex trait, which is affected by multiple factors and displays a strong phenotypic plasticity. Previous investigations suggested that rice tiller angle is strongly related to plant gravitropic responses. Zhang *et al. *(2018) described a core regulatory pathway controlling rice tiller angle: *HSFA2D* regulates auxin asymmetric distribution by regulating expression level of *LA1*, which in turn induce the asymmetric expression of *WOX6* and *WOX11* and thus modulates rice shoot gravitropism and tiller angle. Our study shows that the OsmiR167a‐*OsARF12/17/25* modules also regulate tiller angle via the auxin‐mediated asymmetric distribution of *WOX6* and *WOX11* (Figure S4). Considering the expression of *OsARF12*/*17*/*25* were induced by exogenous IAA treatment (Figure S2), we further examined the expression levels of *OsARF12/17/25* at the lower and upper sides of the shoot based upon gravistimulation under light (Figure S5). It seems that the asymmetric distribution of auxin by gravistimulation have a feedback regulation on the asymmetric expression of *OsARF12/17/25* in the shoot base. Since the expression of *OsARF12/17/25* were suppressed in *hsfa2d* or *la1*, while the expression of *HSFA2D* and *LA1* were increased in *osarf12/osarf17* or *osarf12/osarf25* plants, we speculate that *HSFA2D‐LA1* can induce the asymmetric distribution of auxin as they response to gravistimulation, while *OsARF12/OsARF17/OsARF25* is required for the asymmetric distribution of auxin, possibly by maintaining the location auxin to a suitable level to response to *HSFA2D‐LA.*

**Figure 7:**
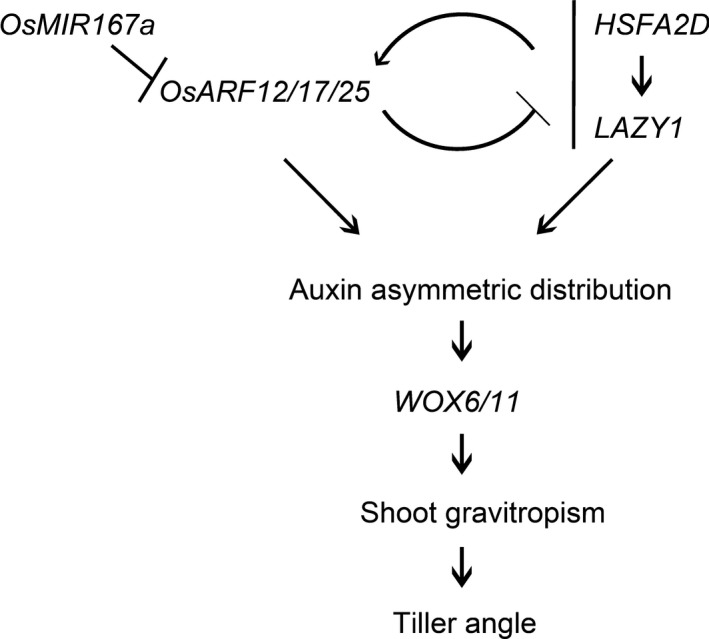
The integrated model controlling tiller angle mediated by OsmiR167a‐*OsARFs* and *HSFA2D*‐*LA1* (Zhang et al., 2018) in rice.

Based on our results, we propose that both OsmiR167a‐*OsARF12/17/25* and *HSFA2D*‐*LA1* could regulate the asymmetric distribution of auxin and subsequently induce the asymmetric expression of *WOX6*/*11* and specify tiller angle (Figure 7). It was well known that auxin regulates cell division and elongation to drive plant growth and development (Woodward and Bartel, 2005). ARF transcription factor family regulates gene expression depend on the distribution of auxin in cells (Chandler, 2016; Li *et al.*, 2016). Our investigation demonstrates that OsmiR167a‐*OsARF12/17/25* regulates the asymmetric distribution of auxin in axillary buds (Figure 5e) and the auxin distribution also resulted in asymmetric expression of *OsARF12/17/25* (Figure S5). Future studies should focus on examining the feedback loop regulation between OsmiR167a‐*OsARF12/17/25* and dose‐sensitive auxin signalling, which might be essential for fine‐tuning tiller angle in rice.

## Methods

### Plant material and growth conditions

Rice plants used in this study were *japonica* (*O. sativa ssp. geng*) variety Zhonghua 11 (ZH11), except *osarf25* in Dongjing background. Rice plants were cultivated in the experimental field of Huazhong Agriculture University in the normal growing season in Wuhan, China (latitude 30.5°N, 15 m above sea level; average daily temperature approximately 28°C).

### Plasmid construction and rice transformation

The genomic sequence containing OsmiRNA167a precursor (http://www.mirbase.org/search.shtml) was cloned into the pU1301 vector, then electroporated into the Agrobacterium tumefaciens strain EHA105, and finally transformed into rice callus to generate miR167a overexpression transgenic plants. The target mimicry strategy was generated as described (Franco‐Zorrilla *et al.*, 2007; Todesco *et al.*, 2010). Three‐nucleotide bulge (CAA) was introduced between the position 10 and position 11 of mature miR167a, and then replaced the 24nt OsmiR399 complementary motif in *OsIPS1* with the modified 24nt of miR167a. The generated artificial target mimics of *MIM167a* were under the control of a maize ubiquitin promoter. To construct the *OsARF* overexpression plasmids, the full‐length coding sequence (CDS) of *OsARF12*, *OsARF17* and *OsARF25* were amplified from the cDNA prepared from ZH11 seedlings and cloned into the pU2301 vector, then electroporated into EHA105 and transformed into ZH11 or *MIR167a‐OE* rice callus. Primers for plasmid construction are listed in Table S4.

### RNA extraction and expression analysis

Total RNA was extracted using TRIzol reagent (Invitrogen, http://www.invitrogen.com/). First‐strand cDNA synthesis was carried out using a reverse transcription kit (Invitrogen). Quantitative RT‐PCR was performed with the first‐strand cDNA as the template. Endogenous ubiquitin transcripts were used to normalize the expression levels. Quantitative RT‐PCR was performed on an ABI 7500 Sequence detection system using SYBR Green. Each set of experiments was repeated three times. The methods for miR167a reverse transcript detection were reported by Varkonyi‐Gasic *et al. *(2007). For the miRNA gel blot analysis, 40 μg total RNA was resolved on a urea‐containing 15% polyacrylamide gel (PAGE), transferred to a Hybond‐N + membrane (Amersham, Arlington Heights, IL) and hybridized with an antisense oligonucleotide probe with a ^32^Pi label as the miR167 probe. All primers used for expression analysis are listed in Table S5.

### RLM‐5′RACE

RNA ligase‐mediated 5′RACE was conducted with the GeneRacer Kit according to the manufacturer's instructions (Invitrogen). Total RNA for RACE was obtained from the young tissues in rice, gene‐specific primers were designed according to each target ARF genes and used for each RACE PCR. The gene‐specific primers of *OsARF6*, *ARF12, OsARF17* and *OsARF25* for the first and second PCR products are OsARFs‐RACE and OsARFs‐RACE‐Nested (Table S6), respectively. The second PCR products were gel purified and subcloned into the pGEM‐T Easy Vector (Promega Corporation MD, USA) for sequencing.

### Collection of mutants and *DR5::GUS/GFP* transgenetic plants

The *osarf12*, *osarf17*, *hsfa2d* and *lazy1* mutants were identified in our T‐DNA insertion mutant library (http://rmd.ncpgr.cn/; Wu *et al.*, 2003). The *osarf25* mutant was previously identified (Qi *et al.*, 2012). Homozygous plants for T‐DNA insertions were identified by PCR‐based genotyping. The primer sequences used for genotyping are listed in Table S7. The *DR5::GUS* and *DR5::GFP* transgenic lines were kindly provided by He Yubing from Huazhong Agricultural University.

### Phytohormone treatments

For exogenous hormone treatment, 7‐day‐old ZH11 seedlings were treated in liquid 1/2 MS medium containing different kinds of phytohormones for 2 h, including ABA (abscisic acid, 10 μM), JA (jasmonic acid, 10 μM), GA3 (gibberellin, 10 μM), BR (brassinolide, 10 μM), IAA (auxin, 10 μM), 6‐BA (6‐benzylaminopurine, 10 μM), NAA (1‐naphthylacetic acid, 10 μM) and Trp (tryptophan, 10 μM), and DMSO as the control. A 1.5‐cm‐long piece of the rice seedling shoot base was harvested for RNA extraction after treatment. For IAA and 1‐naphthylphthalamic acid (NPA) treatment, 7‐day‐old ZH11 seedlings were transferred to hydroponic solution containing 1 μM IAA or 0.2 μM of NPA for 40 days.

### IAA measurement and auxin transport assay

The young axillary buds and roots with about 5‐cm length were collected for IAA content analysis. IAA extraction and measurement were performed according to previous reports (Liu *et al.*, 2012b). The polar auxin transport assays were performed using 7‐day‐old dark‐grown coleoptiles, as described previously with minor modifications (Li *et al.*, 2007). The radioactivity was counted by a liquid scintillation counter (1450 MicroBetaTriLux, PerkinElmer, Boston, MA, USA).

### Shoot gravitropism assay

The gravity response was measured according to the method described previously (Zhang *et al.*, 2018). Three‐day‐old rice seedlings were grown in 1/2 MS medium after being dehusked and surface sterilized. The seeds were then grown at 28°C for 2 days (16 h light and 8 h dark) and were subsequently reoriented by 90° from the vertical for 1 day (gravistimulation treatment). The seedling shoots were analysed, and the shoot curvature was recorded.

### Accession numbers

All related sequence and accession numbers are listed in Table S8.

## Conflict of interest

The authors declare no competing financial interests.

## Author contributions

C.W conceived and supervised this research. Y.L. and J.L. performed most of the experiments. Z.C. and Y.W. generated some transgenetic plants. Y.Q. characterized some mutant lines. C.W. and Y.L. wrote the manuscript. All authors read and approved the final manuscript.

## Supporting information


**Figure S1 **Inactivation of miR167a activity by target mimicry.
**Figure S2** *OsARF12*, *OsARF17* and *OsARF25 *response to auxin treatment.
**Figure S3** Map‐based cloning of *LAZY1*.
**Figure S4** miR167 and *OsARF12/17/25* regulate *WOX6* and *WOX11* asymmetric expression upon gravistimulation under light.
**Figure S5** The expression levels of *OsARF12/17/25 *at the lower and upper sides of the shoot base upon gravistimulation under light.
**Table S1** Bioinformatics analysis of *MIR167* gene family in *Oryza sativa *and *Arabidopsis thaliana* based on miRU prediction.
**Table S2** Putative target genes of miR167 in rice based on CSRDB (Cereal small RNAs Database) (http://sundarlab.ucdavis.edu/smrnas/) prediction.
**Table S3** Primers of PCR‐based molecular markers for *LAZY1*.
**Table S4** Primer sequences for plasmid construction.
**Table S5** Primer sequences used for miR167 and gene expressions analysis.
**Table S6** Primer sequences for RLM‐RACE.
**Table S7** Primer sequences used for genotyping of T‐DNA mutants.
**Table S8** Accession numbers of all related genes in this study.
