# Supplementary material for: OsmiR167a‐targeted auxin response factors modulate tiller angle via fine‐tuning auxin distribution in rice
Source: Plant Biotechnol J. 2020 Mar 4;18(10):2015–26. doi: 10.1111/pbi.13360 (PMC7540336; doi:10.1111/pbi.13360)
Supplement: Supplementary file 1 — Figure S1 Inactivation of miR167a activity by target mimicry. Figure S2 OsARF12, OsARF17 and OsARF25 response to auxin treatment. Figure S3 Map‐based cloning of LAZY1. Figure S4 miR167 and OsARF12/17/25 regulate WOX6 and WOX11 asymmetric expression upon gravistimulation under light. Figure S5 The expression levels of OsARF12/17/25 at the lower and upper sides of the shoot base upon gravistimulation under light. Table S1 Bioinformatics analysis of MIR167 gene family in Oryza sativa and Arabidopsis thaliana based on miRU prediction. Table S2 Putative target genes of miR167 in rice based on CSRDB (Cereal small RNAs Database) (http://sundarlab.ucdavis.edu/smrnas/) prediction. Table S3 Primers of PCR‐based molecular markers for LAZY1. Table S4 Primer sequences for plasmid construction. Table S5 Primer sequences used for miR167 and gene expressions analysis. Table S6 Primer sequences for RLM‐RACE. Table S7 Primer sequences used for genotyping of T‐DNA mutants. Table S8 Accession numbers of all related genes in this study. [file PBI-18-2015-s001.docx]

**Supporting Information**

**Article title:** OsmiR167a-targeted auxin response factors modulate tiller angle via fine-tuning auxin distribution in rice

**Authors:** Yan Li, Jiali Li, Zhihui Chen, Yi Wei, Yanhua Qi and Changyin Wu

The following Supporting Information is available for this article:

**Figure S1** Inactivation of miR167a activity by target mimicry.

**Figure S2** *OsARF12*, *OsARF17* and *OsARF25* response to auxin treatment.

**Figure S3** Map-based cloning of *LAZY1*.

**Figure S4** miR167 and *OsARF12/17/25* regulate *WOX6* and *WOX11* asymmetric expression upon gravistimulation under light.

**Table S1** Bioinformatics analysis of *MIR167* gene family in *Oryza sativa* and *Arabidopsis thaliana* based on miRU prediction.

**Table S2** Putative target genes of miR167 in rice based on CSRDB (Cereal small RNAs Database) (<http://sundarlab.ucdavis.edu/smrnas/>) prediction.

**Table S3** Primers of PCR-based molecular markers for *LAZY1*.

**Table S4** Primer sequences for plasmid construction.

**Table S5** Primer sequences used for miR167 and gene expressions analysis.

**Table S6** Primer sequences for RLM-RACE.

**Table S7** Primer sequences used for genotyping of T-DNA mutants.

**Table S8** Accession numbers of all related genes in this study.


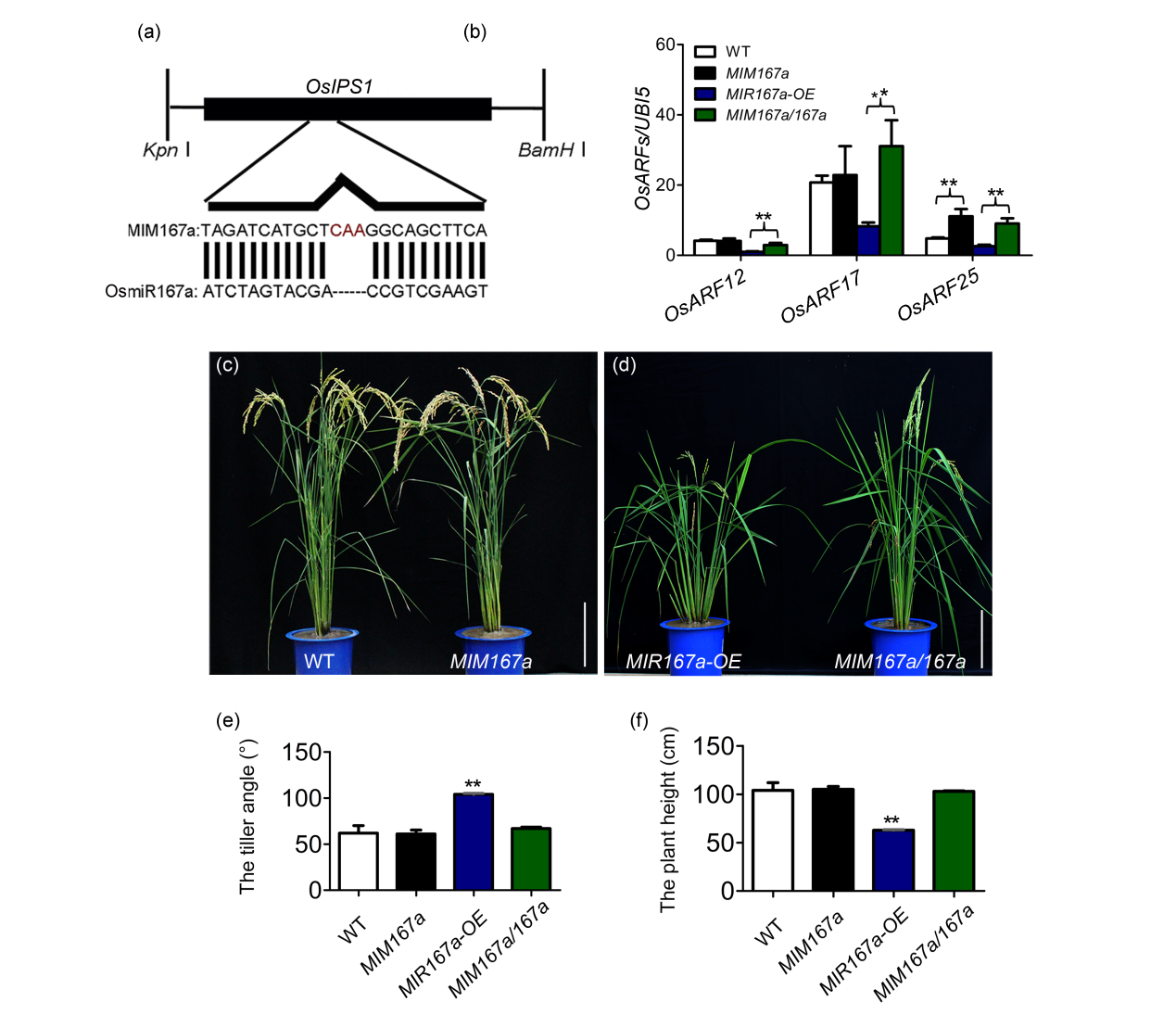


**Figure S1** Inactivation of miR167a activity by target mimicry. (a) Schematic diagram of *MIM167a*. The vertical lines represent the nucleotides of MIM167a are same with OsmiR167a and red show the mismatched nucleotide. (b) The expression of *OsARFs* in wild type (WT), *MIR167a-OE*, *MIM167a* and *MIM167/167a* plant. Rice *UBIQIUTIN5* was used as an internal control. Data are presented as means ± SE (n=3). Significant at **P< 0.01. (c, d) Phenotype of *MIM167a* transformed into WT (c) and *MIR167a-OE* (d) background. Bars=20 cm. (e-f) Comparison of the tiller angle (e) and plant height (f) between WT and *MIR167a-OE* transgenic lines. Data are shown as means ± SE (n=10). Significant at **P< 0.01


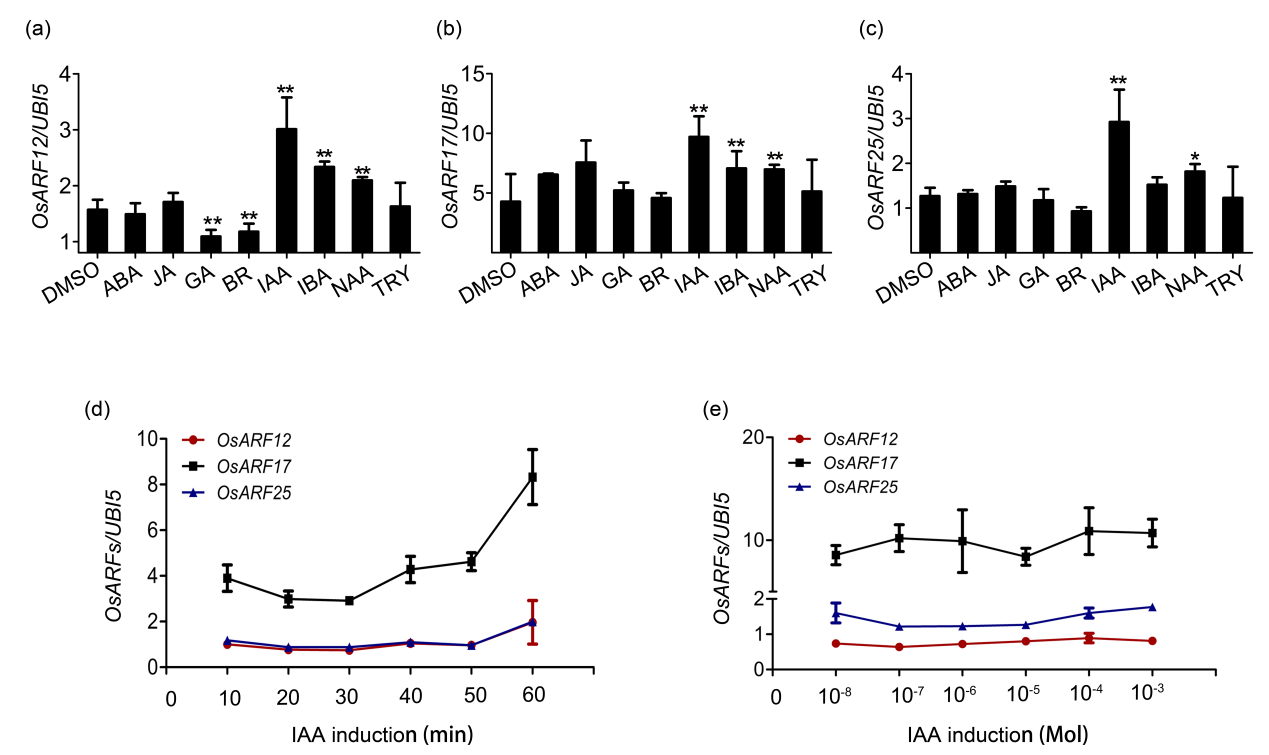


**Figure S2** *OsARF12*, *OsARF17* and *OsARF25* response to auxin treatment. (a-c) Expression analysis of *OsARF12*, *OsARF17* and *OsARF25* under different phytohormone treatments. (d) Time-course response of *OsARF12*, *OsARF17* and *OsARF25* to 10μM IAA treatment. (e) Expression levels of *OsARF12*, *OsARF17* and *OsARF25* under different concentrations IAA treatment. Rice *UBIQIUTIN5* was used as an internal control. Data are presented as means ± SE (n=3). Significant at *P<0.05, **P<0.01

**
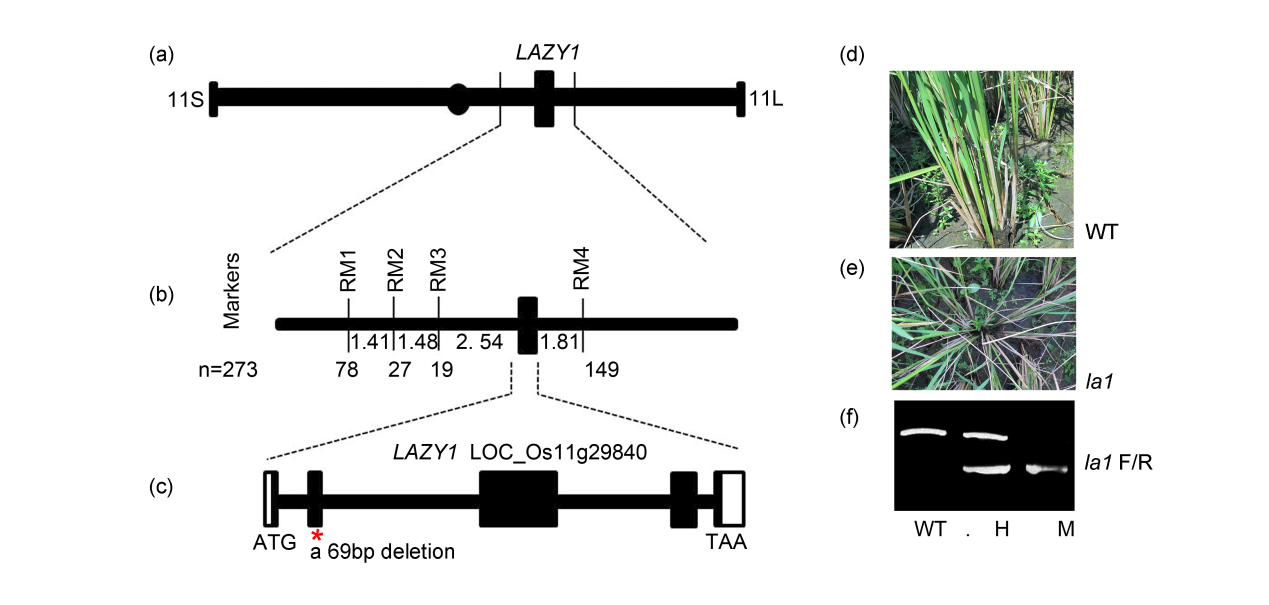
Figure S3** Map-based cloning of *LAZY1*. (a, b) Linkage map of *LAZY1* on chromosome 11. The number of recombinants between the molecular marker and *LAZY1* is indicated. (c) Gene structure of *LAZY1*. The black boxes represent the exons and lines between the boxes represent introns. (d, e) The increased tiller angle of WT (d) and *lazy1* (e) when grew in field. (f) Genotyping of *lazy1* by PCR analysis. Primers of PCR-based molecular markers developed in this study are listed in Table S7.

**
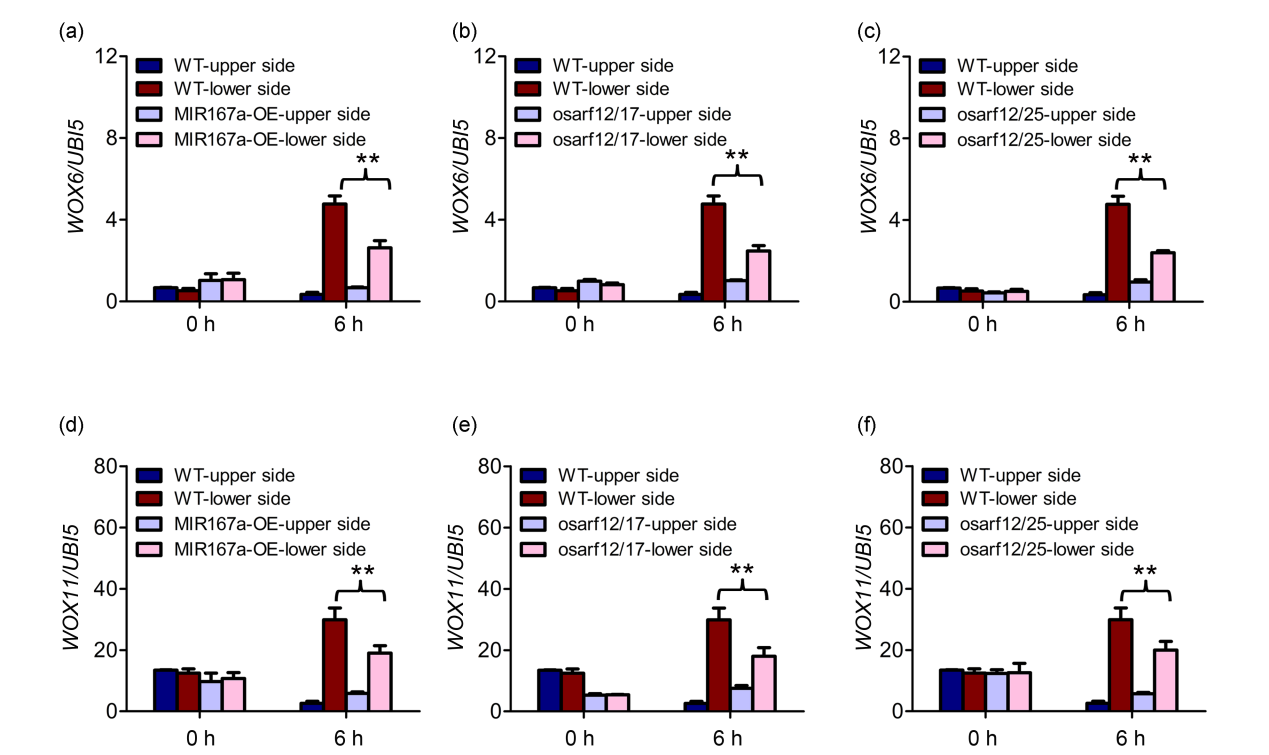
Figure S4** miR167 and *OsARF12/17/25* regulate *WOX6* and *WOX11* asymmetric expression upon gravistimulation under light. (a-f) Expression levels of *WOX6* (a-c) and *WOX11* (d-f) at the lower and upper sides of the shoot base. Values represent means ± SEM (n=3). Significant at **P<0.01

Table S1 Bioinformatics analysis of *MIR167* gene family in *Oryza sativa* and *Arabidopsis thaliana* based on miRU prediction.

| IDs | Lengths of Stem-loop structure (nt) | Genome Contexts | Mature Sequence of miR167 |
| --- | --- | --- | --- |
| *Osa-MIR167a* | 141 | Chr12:25476808-25476948 [+] | UGAAGCUGCCAGCAUGAUCUA |
| *Osa-MIR167b* | 163 | Chr3:30546928-30547090 [-] | UGAAGCUGCCAGCAUGAUCUA |
| *Osa-MIR167c* | 163 | Chr3:33130619-33130781 [+] | UGAAGCUGCCAGCAUGAUCUA |
| *Osa-MIR167d* | 110 | Chr7:4166295-4166404 [-] | UGAAGCUGCCAGCAUGAUCUG |
| *Osa-MIR167e* | 273 | Chr2:3742241-3742513 [-] | UGAAGCUGCCAGCAUGAUCUG |
| *Osa-MIR167f* | 113 | Chr10:14723044-14723156 [-] | UGAAGCUGCCAGCAUGAUCUG |
| *Osa-MIR167g* | 82 | Chr3:3347682-3347763 [+] | UGAAGCUGCCAGCAUGAUCUG |
| *Osa-MIR167h* | 120 | Chr12:25480618-25480737 [+] | UGAAGCUGCCAGCAUGAUCUG |
| *Osa-MIR167i* | 201 | Chr6:27674749-27674949 [+] | UGAAGCUGCCAGCAUGAUCUG |
| *Osa-MIR167j* | 160 | Chr1:32686068-32686227 [-] | UGAAGCUGCCAGCAUGAUCUG |
| *Ath-MIR167a* | 138 | Chr3: 8108072-8108209 [+] | UGAAGCUGCCAGCAUGAUCUA |
| *Ath-MIR167b* | 109 | Chr3: 23406168-23406276 [+] | UGAAGCUGCCAGCAUGAUCUA |
| *Ath-MIR167c* | 160 | Chr3: 1306622-1306781 [-] | UAAGCUGCCAGCAUGAUCUUG |
| *Ath-MIR167d* | 377 | Chr1: 11137539-11137915 [+] | UGAAGCUGCCAGCAUGAUCUGG |

The mature sequence of miR167 was absorded from the miRU web program (<http://www.mirbase.org/index.shtml>). The miR167 of Arabidopsis (TAIR10) and rice (MSU7) were used for bioinformatics analysis. The [+]/[-] represent the direction of the gene on the chromosome.

Table S2 Putative target genes of miR167 in rice based on CSRDB (Cereal small RNAs Database) (<http://sundarlab.ucdavis.edu/smrnas/>) prediction.

| Transcript ID | Predicted functions | score | Mismatch | |
| --- | --- | --- | --- | --- |
| LOC_Os04g57610 | Auxin response factor 12 | 30 | 3 | |
| LOC_Os02g06910 | Auxin response factor 6 | 28 | 4 | |
| LOC_Os06g46410 | Auxin response factor 17 | 28 | 4 | |
| LOC_Os12g41950 | Auxin response factor 25 | 28 | 4 |  |

Table S3 Primers of PCR-based molecular markers for *LAZY1.*

| Name | Primer sequences (5'-3') |
| --- | --- |
| RM1-F | AGCGCATAGGCCTTTCCATTAGC |
| RM1-R | CGTGTTCGTGGGTCTTGTTATGC |
| RM2-F | GCGGACACACCAGAGAATAAGC |
| RM2-R | GTGCTGTCCTGTCCTTGAATCC |
| RM3-F | CTCCCTTCTTCTGCTGAGACACC |
| RM3-R | CAAGCTACTCATTGGGCTCATCC |
| RM4-F | GTAAACCCTAACCGCCTGCTTGG |
| RM4-R | ATTGTCGAGGCGTTTCACAAGG |
|  |  |

Table S4 Primer sequences for plasmid construction.

| Name | Primer sequences(5'-3') |
| --- | --- |
| MIR167a-OE-F | CTTGGTACCTTCTTCCATCAAGCTCACTCA |
| MIR167a-OE-R | CTTGGATCTGCAACAACTTACCCTGTACC |
| OsIPS1-OE-F | CCCTGCCTTCATACGCTATT |
| OsIPS1-OE-R | ATTGCCAAATGTTTGAACGA |
| MIM167a-I | ATTGAAGCTGCCTTGAGCATGATCTACCTTAGTAGAGGTAAAAGTC |
| MIM167a-II | GGTAGATCATGCTCAAGGCAGCTTCAATTATTCGGTGGATGTCTGT |
| OsARF12-OE-F | AAAGGTACCCGCTTAAATGCCACAAACCTAAT |
| OsARF12-OE-R | AAAGGATCCGGAAGACTCGATGAAACATGAAAA |
| OsARF12-immune-F | CTCCCTGTCGACGAATACTAGTTGCCAACCAGACCTATCAGGGTCGTCCAATTGACCCTT |
| OsARF12-immune-R | AAGGGTCAATTGGACGACCCTGATAGGTCTGGTTGGCAACTAGTATTCGTCGACAGGGAG |
| OsARF17-OE-F | AAAGGTACCATGGAGTCTCAGATCCCCAATTATC |
| OsARF17-OE-R | AAAGGATCCTCAGAATTCAACTGAGCCGACAGAT |
| OsARF17-immune-F | ATCCTCTCGGTCGACGAATACTAGTTGCCAACCAGACCTCAAAGGGTTTTCCAACTGGC |
| OsARF17-immune-R | GCCAGTTGGAAAACCCTTTGAGGTCTGGTTGGCAACTAGTATTCGTCGACCGAGAGGAT |
| OsARF25-OE-F | AAAGGTACCTGATTGGTTTCCAAGTTTGATTG |
| OsARF25-OE-R | AAAGGATCCGGTGCTTAGTTTGGATTCTGGTC |
| OsARF25-immune-F | CATTTTCCCGGTCGACAAATACTAGTTGCCATCCAGACCTTACAGGGTCCTCCAGTTG |
| OsARF25-immune-R | CAACTGGAGGACCCTGTAAGGTCTGGATGGCAACTAGTATTTGTCGACCGGGAAAATG |

Table S5 Primer sequences used for miR167 and gene expressions analysis.

| **Name** | **Primer sequences (5'-3')** |
| --- | --- |
| MIR167a ST-RT primer | GTCGTATCCAGTGCAGGGTCCGAGGTATTCGCACTGGATACGACTAGATC |
| MIR167a Forward primer | CTAATGAAGCTGCCAGCATG |
| ST-R | GTGCAGGGTCCGAGGT |
| U6-F | TACAGATAAGATTAGCATGGCCCC |
| U6-R | GGACCATTTCTCGATTTGTACGTG |
| miR167a-antisense probe | TGAAGCTGCCAGCATGATCTA |
| qRT-OsARF1-F | ACCGCTAGAACTCGCATAAA |
| qRT-OsARF1-R | AGTGAAAGCCACCTTCCATT |
| qRT-OsARF6-F | TTGGAAGGTTGCTCGACATC |
| qRT-OsARF6-R | TCCTGCGGCGAAAGTATCT |
| qRT-OsARF8-F | TACCATCACAACCACCCGTCC |
| qRT-OsARF8-R | AGGTGGGTAAGGTGGAGATCTGA |
| qRT-OsARF10-F | TGGTTTCCCTTTCCTCCCCTTC |
| qRT-OsARF10-R | CATCAGATTCGGCTGCAGGTTG |
| qRT-OsARF11-F | AGTGCGAAGCCGATGAAA |
| qRT-OsARF11-R | CTGCCAGGATGCTCCAAT |
| qRT-OsARF12-F | GTTGGGAGGTCGTTGGACATAA |
| qRT-OsARF12 | AAGCACATCATTCTCCCTGTCG |
| qRT-OsARF13-F | CTCCTCTTCCCATGGGGCTAA |
| qRT-OsARF13-R | GTTATGCCTGGCTCCCTGAATG |
| qRT-OsARF14-F | AGCGAAATCACGAGCAAC |
| qRT-OsARF14-R | TCAGGGAGAAGCCAAACA |
| qRT-OsARF17-F | TTTACAAATCGGGAACCTATGG |
| qRT-OsARF17-R | TTTATGCAGGAGACGCTATTCA |
| qRT-OsARF18-F | CATCCACCTTGCTCCATTCTC |
| qRT-OsARF18-R | ATGCCTGGCTCCCTGTATG |
| qRT-OsARF22-F | CCCATTTGAGGGTCATCTTCTG |
| qRT-OsARF22-R | GTAAACCAAATTGAGCATGCCTG |
| qRT-OsARF23-F | GCTATGAAGAGTTGATTGCTGA |
| qRT-OsARF23-R | GATCTTGTGAACCATGTCGC |
| qRT-OsARF25-F | TGACATCTCCAGATTCAGCAGC |
| qRT-OsARF25-R | CGTCTCCACCACGAACCAA |
| qRT-OsPIN1-F | ATCCGCAACCCCAACACCTA |
| qRT-OsPIN1-R | CGGCATCTCGAAGTTCCACC |
| qRT-OsIAA4-F | ACAAGTTCTTCTCCCACTTCACCA |
| qRT-OsIAA4-R | TTCACCGCGTCCACCAGCTTCCTC |
| qRT-OsIAA21-F | GATTGGATGCTTGTTGGTGA |
| qRT-OsIAA21-R | TCTTGTCAGTGGCTCTTGGA |
| qRT-OsGH3.1-F | CGGGAACAAGCAATGGAACA |
| qRT-OsGH3.1-R | CAGATCATCACCCTCTAGCTTCAA |
| qRT-OsGH3.4-F | CGTCACGTACGAGGACATCC |
| qRT-OsGH3.4-R | GCTCCATCTCCTCCTCTATCG |
| qRT-OsGH3.8-F | CCACCCCGTCTCCGAGTTC |
| qRT-OsGH3.8-R | TTGTCAAGCCCAGGCACATACA |
| qRT-OsGH3.13-F | TGTGTAATGTCAAACGTTGCTCAT |
| qRT-OsGH3.13-R | TGATTCATAAAGAACACTGCTCGTATT |
| qRT-PROG1-F | GAGCTCGGCCTCTAGATCAT |
| qRT-PROG1-R | ATCATGGTGATCGTGGAGAT |
| qRT-TAC3-F | CTCACATTAACTTCCAGCACCAA |
| qRT-TAC3-R | TCGGTGGATGATGAGGAGAG |
| qRT-PIN2-F | CGATACTCTACTATGTGCTTCTTGG |
| qRT-PIN2-R | TTCCTCTAGAAACCTAGAATCCTATTT |
| qRT-ONAC106-F | TCATCAGCTCACCATCAC |
| qRT-ONAC106-R | GGTCATCTGACGACGAAG |
| qRT-MADS57-F | CAGATTATGTTGTCGGATGCTC |
| qRT-MADS57-R | AAAGCAATAGAGAGTAAGCAGGGT |
| qRT-IAA20-F | TGGCGGATATGTGAAGGTGAA |
| qRT-IAA20-R | TATGAGCCGAGGATGGACAAG |
| qRT-TIL1-F | CGCCGTGTACGTTAATCG |
| qRT-TIL1-R | ACATCTCCCAAATCACCAAATAATC |
| qRT-FUCT-F | GGAGTCTGCTGTGCTTGCTA |
| qRT-FUCT-R | ACTGGTATAATGCCTGTCGTTGTG |
| qRT-TAC1-F | CACAAGAAGCCAACAAAACC |
| qRT-TAC1-R | GCATTGCTACGTCCATTGA |
| qRT-EPR1-F | GAGAGTGACGGTGTGAAG |
| qRT-EPR1-R | ATCGCTCCAAGTGATTCC |
| qRT-LPA1-F | GCGTATGTATGTAAAGCAAG |
| qRT-LPA1-R | GAAACGACCTACGAAACTAC |
| qRT-CRCT-F | TTCTGGGTGCCTCAACTCA |
| qRT-CRCT-R | AACGCTGTCTCAAAGTCCAATC |
| qRT-AGPL1-F | TTGATTCCACATGGCAGAGAAC |
| qRT-AGPL1-R | GTTGCTGCTGCTACTTCACT |
| qRT-HSFA2D-F | CAGCAGGCACTTGGCACC |
| qRT-HSFA2D-R | TTCTTGTCACGCTTTAGCCTGT |
| qRT-LAZY1-F | GAGATGAACGGCAACAAG |
| qRT-LAZY1-R | TTCCAGCACCAAGTAGTC |
| qRT-WOX6-F | TCCAATAGACTTGCGAGCCAT |
| qRT-WOX6-R | GCATTAGGATTCCATAGTCGTT |
| qRT-WOX11-F | CGGTGTTCATCAACGGAGTG |
| qRT-WOX11-R | TCTGGAGAGAATGGAGGAGGAT |
| qRT-Ubiquitin 5-F | AACCAGCTGAGGCCCAAGA |
| qRT-Ubiquitin 5-R | ACGATTGATTTAACCAGTCCATGA |

Table S6 Primer sequences for RLM-RACE.

| Name | Primer sequences(5'-3') |
| --- | --- |
| OsARF6-RACE | GCGGCACTACAGCACTACTCA |
| OsARF6-RACE-Nested | CAGAACTGGACGCCTAACG |
| OsARF12-RACE | AGTGGACTGCCGTGCGAGTTACAAA |
| OsARF12-RACE-Nested | GCACCGAATAGGTAACAGAGAGT |
| OsARF17-RACE | CACAGGATTAGTGTTTCATGGAACA |
| OsARF17-RACE-Nested | TCTTTCAGTTCCAGGTCTC |
| OsARF25-RACE | GACTGAGTTGCTACTGATTCGGACA |
| OsARF25-RACE-Nested | AGGGTGCTTAGTTTGGATTCTGGTC |

Table S7 Primer sequences used for genotyping of T-DNA mutants.

| Name | Primer sequences(5'-3') |
| --- | --- |
| HYG-F | ATTTGTGTACGCCCGACAGT |
| HYG-R | ATTCCGGAAGTGCTTGACAT |
| osarf12-F | CTTCAGGACAGATACCGTGGAT |
| osarf12-R | GGGAGGTCGTTGGACATAAC |
| osarf17-F | CCTGTTATCGTGCCCATGTATC |
| osarf17-R | TGTTCCCTCCGCTGGTTTG |
| osarf25-F | CGGAAGAGGGATAACAGTC |
| osarf25-R | TTAGTTCTGTCAGGTTGCG |
| hsfa2d-F | ATGTTTTGTGCCACATCGTG |
| hsfa2d-R | TCCTGCTGGTGAATCAACTG |
| T-DNA L1 | AATCCAGATCCCCCGAATTA |
| la1-F | TGATAAGACGGACGGTCAAACAT |
| la1-R | ACGAGCGCCACCTTCTCGTC |

Table S8 Accession numbers of all related genes in this study.

| **Name** | **Species** | **Accession Numbers** | **Database** |
| --- | --- | --- | --- |
| ARF1 | *Oryza sativa* | LOC_Os01g13520 | MSU-RGAP |
| ARF6 | *Oryza sativa* | LOC_Os02g06910 | MSU-RGAP |
| ARF8 | *Oryza sativa* | LOC_Os02g41800 | MSU-RGAP |
| ARF10 | *Oryza sativa* | LOC_Os04g43910 | MSU-RGAP |
| ARF11 | *Oryza sativa* | LOC_Os04g56850 | MSU-RGAP |
| ARF12 | *Oryza sativa* | LOC_Os04g57610 | MSU-RGAP |
| ARF13 | *Oryza sativa* | LOC_Os04g59430 | MSU-RGAP |
| ARF14 | *Oryza sativa* | LOC_Os05g43920 | MSU-RGAP |
| ARF17 | *Oryza sativa* | LOC_Os06g46410 | MSU-RGAP |
| ARF18 | *Oryza sativa* | LOC_Os06g47150 | MSU-RGAP |
| ARF22 | *Oryza sativa* | LOC_Os10g33940 | MSU-RGAP |
| ARF23 | *Oryza sativa* | LOC_Os11g32110 | MSU-RGAP |
| ARF25 | *Oryza sativa* | LOC_Os12g41950 | MSU-RGAP |
| GH3.1 | *Oryza sativa* | LOC_Os01g57610 | MSU-RGAP |
| HSFA2D | *Oryza sativa* | LOC_Os03g06630 | MSU-RGAP |
| LAZY1 | *Oryza sativa* | LOC_Os11g29840 | MSU-RGAP |
| LPA1 | *Oryza sativa* | LOC_Os03g13400 | MSU-RGAP |
| PROG1 | *Oryza sativa* | LOC_Os07g05900 | MSU-RGAP |
| TAC1 | *Oryza sativa* | LOC_Os09g35980 | MSU-RGAP |
| TAC3 | *Oryza sativa* | LOC_Os03g51660 | MSU-RGAP |
| WOX6 | *Oryza sativa* | LOC_Os03g20910 | MSU-RGAP |
| WOX11 | *Oryza sativa* | LOC_Os07g48560 | MSU-RGAP |

MSU-RGAP database: <http://rice.plantbiology.msu.edu/>
